# Walking at the edge: How tempo-spatial nexus forms HRM practices in project-based organizations

**DOI:** 10.3389/fpsyg.2023.974117

**Published:** 2023-02-09

**Authors:** Ehsan Samimi

**Affiliations:** School of Business & Economics, Freie Universität Berlin, Berlin, Germany

**Keywords:** project-based organizations, temporary organizing, organizational practices, temporality, human resource management

## Abstract

**Introduction:**

Research has scrutinized the role of different HRM practices in Project-based Organizations (PBOs) mostly in terms of project success and articulated the challenges of traditional HRM to reconcile with the project context. Nevertheless, HRM practices have been addressed less in PBOs with a practice-based research lens. Particularly, the role of tempo-spatial nexus in shaping such practices in this organizational form has been under-researched, although PBOs provide a very suitable context for doing so.

**Methods:**

Drawing upon a comparative case study in the oil and gas industry of Scotland and adopting a practice-based approach, this research aims to shed light on how HRM practices are shaped and re-shaped in the project-based context. The study, specifically, scrutinizes the role of temporality and space in the formation, adoption and adaptation of HRM practices in these organizational forms.

**Results and discussion:**

The findings reveal that project characteristics, specifically their duration, size and technical properties, induce different temporalities that along with different work locations and inter-organizational relationships, impact HRM practices as a threefold structure.

## Introduction

Although the role of the “human factor” in project management is uncontested (Strain and Preece, 1999), only some scholars pay attention to the role of human resources in projects and PBOs (Keegan et al., 2018; Samimi and Sydow, 2021). While, some highlight the importance of human resources in terms of resource allocation (Engwall and Jerbrant, 2003; Winch, 2014) others investigate the role of HRM practices mostly in terms of project efficiency and success (Söderlund and Bredin, 2006; Turner et al., 2008; Huemann, 2010, 2015; Keegan et al., 2018; Samimi and Sydow, 2021). What is more, HRM research points to the difficulties and challenges of traditional HRM practices and configurations in attempting to reconcile with specific features of projects and project-based organizations (Söderlund and Bredin, 2006; Huemann, 2010, 2015; Keegan et al., 2018; Samimi and Sydow, 2021). In this sense, research addresses how HRM practices and or concepts may be different in temporary organizations in comparison with traditional, more long-lasting organizations (*cf.*, Keegan et al., 2012; Ferreira et al., 2013; Nuhn et al., 2016; Keegan and Den Hartog, 2019). For instance, Nuhn et al. (2016) show how workers’ turnover intentions in temporary organizations are different from more permanent organizations, and Keegan et al. (2012) reveal how HRM practices in PBOs, are executed beyond the line managers, mostly in collaboration with project managers in this organizational form.

Furthermore, research in both fields of HRM and project management, has also addressed particular HRM practices in the project context and refers to their peculiarities and differences in this context (Keegan and Den Hartog, 2019; Samimi and Sydow, 2021). But the extant research does not scrutinize how these HRM practices are formed in the project context and how contextual elements affect this formation. In the other word, it less clear why HRM practices are different in the project context. Given all this, there are still many unknowns about the formation, adoption and adaptation of these practices embedded in the project-based context (Watson, 2004; Björkman et al., 2014; Gill, 2018) or temporary forms of organizations more generally. Furthermore, while practices, as recurrent situated organizational actions, are shaped in time and space (Giddens, 1984; Schatzki, 2006), it is still not clear how exactly the tempo-spatial nexus in a project-based context contributes to the shaping of organizational practices in general and HRM in particular. While temporary organizational forms, particularly project-based forms (Hobday, 2000), are fruitful grounds to study the effects of time and space in shaping organizational practices, this is surprising.

The combination of permanency and temporariness that characterizes PBOs creates a unique “semi-temporary” (Bakker et al., 2016) or “temporal hybrid” (Samimi and Sydow, 2021) organizational context, where the tension between permanent and impermanent parts, the so-called permanent-temporary dichotomy (Sahlin-Andersson and Söderholm, 2002; Winch, 2014; Sydow and Windeler, 2020), increases the PBOs’ complexity. In the case of HRM, this hybrid form creates difficulties for implementing traditional HRM practices and configurations that are more suited to more permanent organizations (*cf.* Samimi and Sydow, 2021). Besides, the different and sometimes contradictory HRM requirements of the project and more permanent parts in PBOs intensify the challenges of HRM in PBOs (Huemann, 2010, 2015; Keegan et al., 2018). Furthermore, not only the permanency–impermanency tension inside PBOs, but also the embeddedness of projects in their more durable contexts and the mutual relationship between projects and their environment (Windeler and Sydow, 2001; Grabher, 2002; Sydow and Staber, 2002; Engwall, 2003; Sydow et al., 2004; Manning, 2008) underline the interplay between the temporary and the permanent and, more broadly, between stability and change (Farjoun, 2010). On this premise, the temporalities of PBOs and their contexts play a pivotal role in shaping their practices, including HRM. However, temporality has received much less attention in the temporary organizing research, including PBOs (Stjerne and Svejenova, 2016; Stjerne et al., 2019; Braun and Lampel, 2020) and its role in shaping HRM practices in different contexts is also overlooked by the HRM literature (Watson, 2004). However, deciphering this role can shed light on how HRM practices can be reconciled with specific features of the project context in PBOs.

In a similar vein, the role of space in shaping organizational practices in general and HRM practices in particular in the project-based context is under-researched (Maaninen-Olsson and Müllern, 2009; van Marrewijk and Smits, 2014). While, for instance, research points to the effects of the spatial setting in managing megaprojects or “boundary spanning dimensions” (Maaninen-Olsson and Müllern, 2009) that should be taken to account in the management of projects, it is less well understood how the spatial aspects of the project shape organizational practices. These effects are also less studied by considering the interaction between spatial and temporal aspects of projects, focusing on the tempo-spatial relationship.

This research sheds light on how HRM practices are formed and adapted in PBOs beyond the blurred boundaries of permanency and temporariness (Stjerne and Svejenova, 2016; Stjerne et al., 2019) and alongside the “actuality” of project-based working and organizing (Cicmil et al., 2006). Particularly, it will elucidate how the different temporalities of PBOs’ actors, in conjunction with the spatial aspects of projects, form organizational practices in this organizational form. In this sense, it draws on social or events time (Jacques, 1982; Nowotny, 1992) and the spatial aspects (Kornberger and Clegg, 2004; Taylor and Spicer, 2007; Maaninen-Olsson and Müllern, 2009; van Marrewijk, 2009; Beech and Macintosh, 2012; van Marrewijk and Smits, 2014; Weinfurtner and Seidl, 2019; Stephenson et al., 2020) in organizations, particularly temporary organizations.

Adopting the social or event time in organizations (Ancona et al., 2001b), organizational actors constantly form and re-form different temporal structures that shape the temporal pace of their ongoing actions (Dahlgren and Söderlund, 2001; Orlikowski and Yates, 2002). In this sense, temporal conceptions, or temporalities are an integral part of recurrent situated actions, and the practices of organizational actors, and are reflected in the “duration, sequence, timing and frequency of organizational activities” (Zerubavel, 1981). This research explores how these temporal structures, in conjunction with spatial features of projects, affect particular organizational practices, i.e., HRM practices, in PBOs. Following this theoretical approach, this research contributes to the discussions on the peculiarities of HRM practices in the project context in the field of HRM (Huemann, 2010, 2015; Keegan et al., 2018; Samimi and Sydow, 2021) and to the ongoing debate on the role of context in shaping organizational practices following the “project-as-practice” approach in project management research (Blomquist et al., 2010). In particular, the research answers the following two questions:

1. How are HRM practices formed in PBOs?
2. How does the tempo-spatial nexus affect the formation of HRM practices in PBOs?

Drawing upon an embedded multiple-case study (Eisenhardt, 1989; Yin, 2008) in the global energy industry, the paper aims at answering these two research questions. The data have been collected with the help of semi-structured interviews with HRM practitioners and project managers, as well as a systemic review of secondary data from two Scottish project-based firms involved in offshore oil and gas projects in the North Sea.

The structure of the paper is as follows. First, I present the theoretical foundation of the research and then, following a description of the research setting and methodology, I describe the cases and explicate the findings. In the last part, I conclude by comparing and discussing the findings and highlighting the main arguments of the research.

## Theory: Practice-based perspectives on HRM and tempo-spatial nexus

### HRM, PBOs and practice-based perspectives

The ‘practice-turn’ in social theory (Schatzki et al., 2001) has diffused the practice-based approach into different fields of management and organization studies, including project management (Blomquist et al., 2010; Clegg et al., 2018) and HRM (Vickers and Fox, 2010; Ripamonti and Scaratti, 2011). While practice-based perspectives follow different theoretical approaches to define and study the practice (Nicolini, 2013), a practice is often defined as a recurrent situated action that is performed by multiple actors (Bourdieu, 1977; Giddens, 1984; Schatzki, 2006). Following the practice theory, some scholars present a ‘project-as-practice’ approach (Manning, 2008; Blomquist et al., 2010; Clegg et al., 2018) that highlights the role of practices in understanding and organizing “project work” (Ekstedt, 2019), which does not always but can make use of temporary work, whether or not supplied by the temporary-help industry (Kalleberg, 2009).

Research on HRM has also adopted such an approach, and some scholars have studied HRM practices *in-situ* (Watson, 2004; Vickers and Fox, 2010; Ripamonti and Scaratti, 2011). For instance, drawing upon the actor-network and community of practice theories and adopting a longitudinal research design, Vickers and Fox (2010) study practices of “managerial resistance, enrolment and counter-enrolment “in two chemical production plants and show how an unofficial network of managers used formal HRM practices against the official strategy of the firm” (p: 899). In a similar vein, Ripamonti and Scaratti (2011) investigate the role of local knowledge in designing and developing assessment practices and show how local knowledge is used in setting up an assessment center in a multinational organization operating in the transport industry. Apart from these few studies that put the situational and contextual actions of organizational actors at the center of their research and study of HRM *in-situ*, the approach has received little attention in HRM research as yet. Consequently, HRM has rarely been studied *via* the practice-based lens specifically, in the project-based context.

This is surprising, as due to their temporal hybrid form, PBOs allow to study how HRM practices, as the “doings and sayings” of organizational actors (Schatzki, 2006) in a temporary system, meet those of a permanent organization and create fruitful ground to explore contextual elements that affect the shaping and adaptation of HRM practices in the project-context.

Although not adopting a practice lens, previous research on HRM in projects/PBOs has underlined the specific role of single or bundles of HRM activities (Ferreira et al., 2013; Raja et al., 2013; Keegan et al., 2018; Keegan and Den Hartog, 2019; Samimi and Sydow, 2021), mostly in terms of the project’s success, and articulated challenges of traditional HRM in PBOs (Söderlund and Bredin, 2006; Huemann et al., 2007; Keegan et al., 2012; Nuhn et al., 2016). For instance, Raja et al. (2013) point to the difficulties of traditional HRM when attempting to reconcile with different contractual arrangements in international projects, and Söderlund and Bredin (2006) elucidate the challenges of developing competencies in the project context. More recently, Keegan and Den Hartog (2019) show how, in comparison with permanent organizations, performance appraisal is implemented differently in PBOs, following the role of employees “in orchestrating the appraisal process, the multiple actors that have input to appraisal including project managers, the distance between employees and their official line managers, and the weak coordinating role of human resource specialists” (p: 217).

While these and other similar studies (*cf.* Keegan et al., 2018; Samimi and Sydow, 2021) shed light on peculiarities of HRM practices in the project context, the practice turn in HRM research raises important questions about HRM practices in project-based forms of organizing: How are HRM practices affected by the PBO’s structure and context? How do actors, in particular project managers, enact HRM practices under such circumstances? How do PBOs deal with the different temporalities induced by actors’ different time perceptions, and de-spread locations of projects for developing and/or changing HRM practices? These questions do not have clear answers in the extant literature yet.

### Tempo-spatial nexus

Time and temporality have been studied in organizations according to different standpoints (Ancona et al., 2001a; Roe et al., 2009). While time inherently involves ambiguity and abstraction that makes it difficult to grasp (Dawson, 2014a; McNeill, 2015), temporary or semi-temporary organizational forms, such as PBOs, create an opportunity to investigate its different aspects and effects on organizations (Ancona et al., 2001a,b; Roe et al., 2009; Slawinski and Bansal, 2015). However, it seems that scholars are not fully utilizing this fruitful ground.

Among different temporary organizational forms, temporal hybrids, particularly PBOs that combine temporariness and permanency in their structures, are suitable contexts to study not only clock time, for instance by addressing the salient role of deadlines, time cycles, schedules, and dates in projects, but also social or event time (Jacques, 1982; Nowotny, 1992; Lee and Liebenau, 1999; Rubin, 2007). Focusing on time as *kairos* can illustrate the prominent role of time in temporary organizational forms such as PBOs, as different temporalities unfold in the practices of the actors in this organizational form. The notion of temporality focuses on how actors reflect on instants and unfold these reflections in their actions (Mead, 1932). On this premise, temporality is an integral part of human agency, and time is constituted through “emergent events” by continual reflection on the past and future as well as assessment of the present time (Emirbayer and Mische, 1998). Thus, organizational actors constantly shape and re-shape different temporal structures that are reflected in the pace, sequence and duration of their actions (Orlikowski and Yates, 2002).

The concept of temporality has received some attention in project management and organizational studies (Clark and Maielli, 2008; Braun and Lampel, 2020; Clegg et al., 2020; Geraldi et al., 2020; de Vaujany et al., 2021). For instance, recently Clegg et al. (2020) pointed to the concept of memory, “as a bridge between the temporalities of organizing that are past and were never intended to endure, and those that are ongoing” (p: 37). Likewise, Geraldi et al. (2020) investigate the temporal tensions of strategic initiative projects in permanent organizations and identify three temporal tensions, i.e., “ambition versus realism when enacting the time horizon, patience versus urgency when enacting the pace, and clock time versus event time when enacting the temporal perspective” (p: 81). Others have also tried to bring temporality into theorizing temporary organizing (Stjerne and Svejenova, 2016; Stjerne et al., 2019; Sydow and Windeler, 2020).

Moreover, organizational and management scholars have addressed the role of spatial settings and space in organizations (Davis, 1984; Kornberger and Clegg, 2004; Elsbach and Pratt, 2007; Taylor and Spicer, 2007; van Marrewijk, 2009; Hua et al., 2010; Beech and Macintosh, 2012; Weinfurtner and Seidl, 2019). Following a narrative review, Taylor and Spicer (2007) categorize research on space in organizational studies into three categories, i.e., “studies of space as distance, studies of space as the materialization of power relations and studies of space as experience” (p: 325). However, research uses different terms, such as space, region, locale, place, workspace and physical environments to study the concept under these labels. There is an ongoing debate about distinguishing between these different terms, for instance by distinguishing between space and place (Casey, 2003). In a similar vein, Elsbach and Pratt (2007) point to the inconsistencies in research on physical environments in organizations and call for a focus on tensions that exist in “the functions that physical environments serve, i.e., esthetic, instrumental, and symbolic functions” (p: 181). More recently, following a process conception of space, Stephenson et al. (2020) review the process studies of organizational space, which challenge the conception of space as a fixed and physical organizational environment and portray a more dynamic picture of the concept. Drawing on an analysis of six reviews of organizational space from 1984 to 2019, they classify five orientations in existent process research on organization space, i.e., “developing, transitioning, imbricating, becoming, and constituting” (p: 797).

Despite these discussions, the topic has not received much attention in project management research (Maaninen-Olsson and Müllern, 2009; van Marrewijk, 2009; van Marrewijk and Smits, 2014), specifically not in research on HRM in the project context (*cf.*, Keegan et al., 2018). However, research on the role of space in HRM is one of the classic topics in this field, under the label of workspace and/or flexible work arrangements (*cf.*, Jooss et al., 2021; Renard et al., 2021). Van Marrewijk and Smits (2014) study the role of project space and spatial settings in managing megaprojects. Adopting an ethnographic and longitudinal design, the authors study two mega infrastructure projects located in two very different geographical locations, the Netherlands and Panama. The results show how different “intersections of spatial design and organizational development; location of project headquarters and spatial distribution of project locations” shape the actions of project practitioners, which results in both “processes of organizational integration and fragmentation” (van Marrewijk and Smits, 2014, p: 78).

Furthermore, practice theories highlight the role of time and space in shaping practices in context (Giddens, 1984; Nicolini, 2013). However, while time and space have been investigated mostly as separate concepts in the organizational context, it is not yet clear how their interaction or, in other words, tempo-spatial nexus affects the shaping of organizational practices, including HRM practices, in project contexts. In one of only few studies that focus on this nexus in projects, Maaninen-Olsson and Müllern (2009) scrutinize the role of space and time in managing high-tech projects. Drawing on a longitudinal case study, they underscore the different dimensions of boundary spanning that should be taken to account in managing projects, and present seven categories, i.e., “physical distance, functional distance, institutional distance, type of sequence, project duration, need for synchronization and rate and tempo” for analyzing them in conjunction with the tempo-spatial relationship.

Drawing on this background, this research contributes to the discussions on time and space in organizations by exploring how the tempo-spatial nexus in the project context shapes particular organizational practices, i.e., HRM practices in PBOs. An understanding of how these practices meet, intermingle or are kept separate in PBOs would be important to find out how the temporality and spatial elements of the project context affect the formation and shaping of organizational actions (Orlikowski and Yates, 2002) in temporary organizations.

## Research methodology

The research utilizes an embedded multiple-case design to investigate the phenomenon. A case study is a suitable research method for explorative studies seeking to scrutinize not only the *why* but in particular the *how* of a certain phenomenon, such as HRM practices in PBOs. Moreover, the case-study method allows for studying “contemporary events” embedded in context as they are happening (Yin, 2008). In addition, the multiple-case study increases the generalizability of the case study research and assists with finding patterns through the “logic of replication” and cross-case comparison (Eisenhardt, 1989). Applying this premise, and aimed toward finding answers to the research questions, this study utilizes an embedded multiple-case study design. Four cases from two project-based oil and gas service companies were selected for the study. The two firms meet the criteria for being a PBO, since for both, delivering projects to their clients is the primary means of value creation and the sole source of income for the organization (Hobday, 2000; Sydow et al., 2004; Bakker et al., 2016). In more detail, three business units embedded in a project-based oil and gas service company and an oil and gas project-based firm were selected as the cases.

Semi-structured interviews were used as the main data-collection technique in accordance with the explorative nature of the study. Besides, a systematic review of secondary data including policies, procedures and other relevant documents is utilized to diversify data sources (Eisenhardt, 1989). Furthermore, the cases were visited physically for a week to observe the actual activities and to collect as much contextual data as possible. In total, 30 interviews in either face-to-face meetings or through Skype were conducted with HR and project practitioners who were involved directly in HRM practices as well as senior business and operations managers. All the interviews were conducted in English. Questions were asked about how HRM practices were formed and developed in the organization. Interviewees reflected on their day-to-day activities in their area of responsibility as well as exceptional events and occasions in their work. All the interviews were recorded and transcribed to analyze them in conjunction with the other sources of data. Table 1 depicts data sources. A case study protocol and database were developed to ensure the reliability and validity of the research (Yin, 2008). Finally, the case report was presented to key informants to validate and discuss the findings (Eisenhardt, 1989; Yin, 2008).

**Table 1:** Data sources.

Source	Number	Description
Interviews	30 (North Sea Group = 15, North Welding Solutions = 15)	HR practitioners *n* = 12; Operation Managers/Managing Director/Business; Unit Manager *n* = 7; Project managers/head of projects/construction managers *n* = 10; Health, Safety and Environment Manager *n* = 1
Secondary documents	14	Company profiles, HR introductory presentation, HR policies and procedures, HR forms, PRF, minutes of HR and project meetings
Observation in days	7	

The dataset was analyzed through an abductive approach (Sætre and Van de Ven, 2021) iterating between an inductive analysis and one based on the practice-based perspective to explore frames and patterns of data in conjunction with the research questions (Charmaz, 2006; Corbin and Strauss, 2014). In response to the research, patterns were explored within and across the cases for several rounds. Initially, 12 categories were identified that, following the cross-case comparison, were summarized into 6 overarching frames (Eisenhardt, 1989). On this premise, temporality, space, relationships with clients, project type, and industry dynamics were identified as the main themes. Moreover, for the aim of this research, the analysis process delved further into overlaps and intersections of identified codes and focused on their interrelationships. For coding the interviews, a sentence or a paragraph of the interview transcripts was related to the codes. Final codes were reviewed in conjunction with observation field notes and secondary data to ensure consistency. The software package for qualitative data analysis, MAXQDA, was utilized both for transcribing the interviews and the coding process. Table 2 illustrates the data structure.

**Table 2:** Coding structure.

Sample of quotes	Primary themes	Aggregate dimensions
*The OMI context is “quite reactive, fast pace and ever-changing*” (NSG HR Manager) *So we have got two recruiters primarily concentrating on offshore recruitment, which is slightly different from when you do onshore recruitment, because they are more permanent there are fewer, a lot fewer transient [workers] in onshore …” (NSG-Training and Competency Management Advisor)*	Temporary/permanent employees, pace/rhythm of actions, time limitations	Temporality
*“…for offshore we receive the [recruitment] request with short notice. It can be very, very, short notice... Offshore does tend to be a bit more reactive…(NWS-Resource Planning Manager)*	Onshore, offshore	Space
*“small short-term repair projects on brownfields while most of the time the operation is ongoing during the execution of projects”* … “*the reactive nature of projects*, *their short durations and intensive time limitations*” (the Head OMI Projects)	Project duration, project size, technical properties	Project type
*“Each course [for the induction] is specific for clients. We have our induction course on the e-learning system”(NSG-HR Advisor)*	Client interventions, client approvals	Clients relationships
*“…through change to the downturn, …within the industry, a lot of companies actually went away from that so they wanted to have the flexibility to be able to take only the key, core overhead people…”(NWS-CEO)*	Oil price, downturns	Industry dynamics

## Research context and case descriptions

The oil and gas industry has a pivotal role in Scotland’s economy. In 2017, it contributed 9.2 billion British Pounds to the economy and supported 135,000 jobs in Scotland and a total of 375,000 jobs in the United Kingdom (Gibb, 2018; Scottish Affairs Committee, 2019). The industry has a vital role in the UK’s energy security, as well. It is forecast that the industry will supply “two-thirds of the UK’s primary energy needs until at least 2035” (Scottish Affairs Committee, 2019, p: 3).

**Figure 1:**
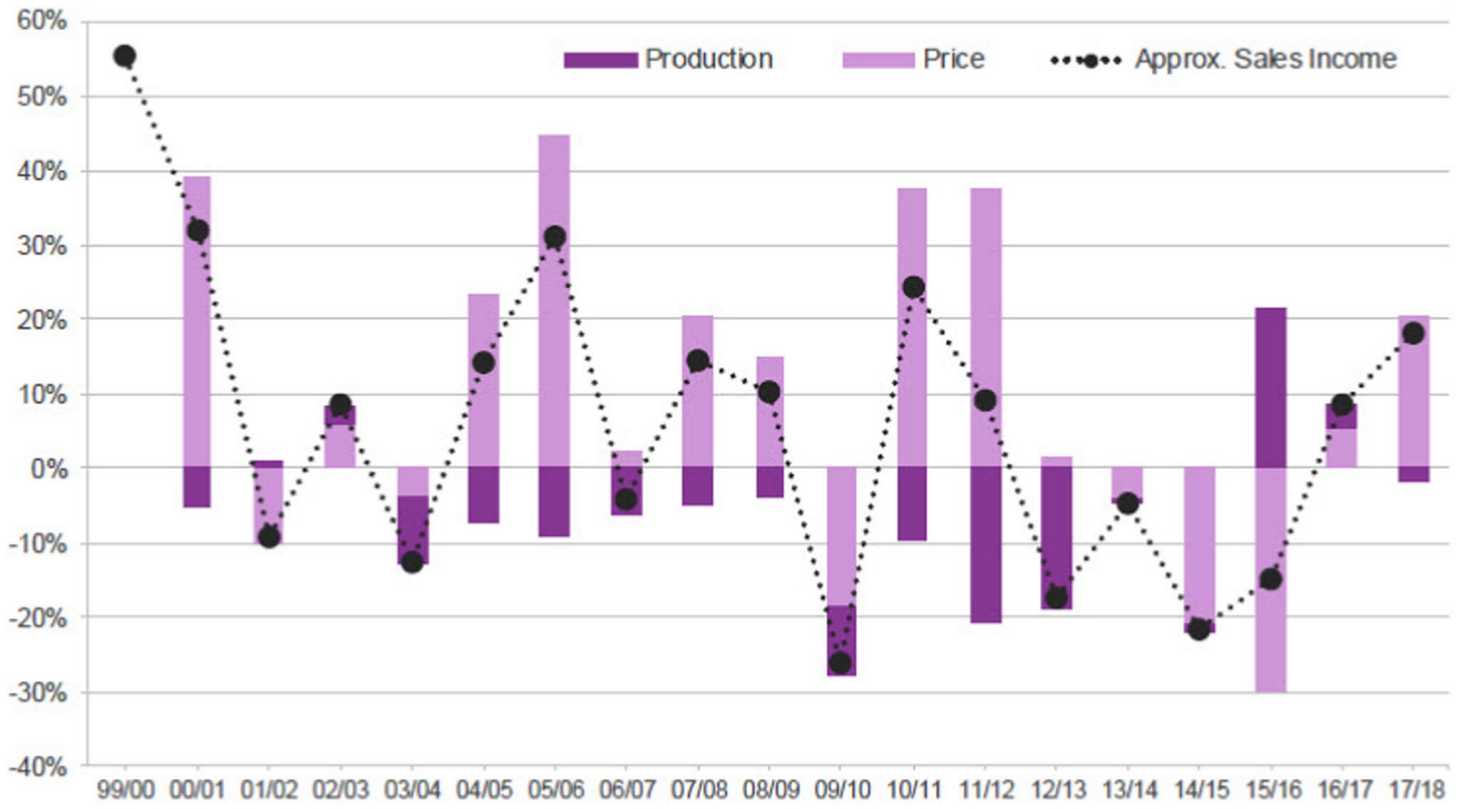
Changes in price, volume and approximate sales income (Scottish Government, 2018: 7).

The North Sea coast is a hub for oil and gas production due to its several, mostly offshore oil and gas fields. Several blue-chip oil and gas companies such as Royal Dutch Shell and British Petroleum own production platforms and/or are involved in the exploration and development of fields in the region. Likewise, there is an abundance of firms in the region involved in providing services to production platforms and projects. Projects play a crucial role throughout the value chain of the oil and gas industry, at different levels both upstream (exploration and production) and downstream (refinery and petrochemicals). Many of these projects are inter-organizational (Jones and Lichtenstein, 2008). Importantly, the industry and, thereby, work in organizational and inter-organizational projects, are strongly affected by oil price fluctuations (Gibb, 2018). Figure 1 depicts the impact of changes in price on the approximate sales income of Scottish oil and gas from 1999 to 2018.

At the time of conducting the empirical fieldwork in 2018, the industry had just passed a challenging downturn. While some positive signs for recovery were being noted, “some key indicators, such as the rate of new well exploration, remained low” (Scottish Affairs Committee, 2019, p: 3).

### The north sea group (cases 1–3)

The North Sea Group (a pseudonym, hereafter NSG), is, like most service firms in the industry, a typical PBO, operating in fabrication, maintenance and marine services projects. NSG provides services to offshore oil and gas production platforms, drilling rigs and special ships used for oil and gas projects in the North Sea. The company has more than 1,300 employees, which can increase to as many as 2,500 employees, depending on the number of active projects. The company was founded in the 1990s and has grown significantly in the last two decades. NSG has dispersed operation facilities in different industrial zones in Scotland.

NSG provides its services *via* three semi-autonomous business units named Offshore Maintenance and Integrity, Fabrication, and Marine Services that are located at the four different locations in Scotland. The three business units, which constitute cases 1–3, are legally independent and have been organized around projects both geographically and technically. While normally, each business unit is involved in a different type of project, they provide services to the other units as well. Each unit has its typical permanent functions such as the commercial, finance and engineering departments, while the HR department located in the offshore maintenance section provides services to the whole group.

Like many PBOs, NSG also utilizes a mixture of temporary and permanent employees. The number of each type varies between business units, depending on project types, i.e., maintenance and marine projects utilize temporary workers the most. The permanent employees have tenured contracts with the firm, while the temporary employees’ category embraces different types of “transient workers,” as they are called by the company. These are either professional freelancers providing their services through an intermediary legal entity (according to the UK’s tax regulations) or agency workers recruited *via* a third party. The HRM function is centralized in the group and supports all business units from its base. Due to geographical distance, many HRM practices are implemented with the assistance of information systems.

#### Case 1: Offshore maintenance and integrity

The Offshore Maintenance and Integrity (hereafter OMI) unit is involved in asset integrity and maintenance projects comprised of repairing and replacing production equipment, modification and enhancement projects, and/or overhaul maintenance services to production platforms or using the technical term, “brownfields.” While some parts of these projects are executed at NSG’s facilities on land, the main part of the projects is executed by technicians and engineers on the offshore platforms. Accordingly, normally people are sent by helicopter daily to platforms to execute the project. “On average 220 people go offshore every week” (Group HR Manager). Each piece of offshore work may last from one to 2 weeks, while each project might take up to 3 months. Most of these rather small projects are based on repair orders from clients and have specific scope and a deadline for delivery. In total, 500 employees work in the OMI unit, of which 80% are transient workers. This includes limited company contractors (freelancers) and agency workers (70 and 10%, respectively). Only 20% of the employees are permanent staff, the classic clientele of in-house HRM. These are mostly project managers, engineers and non-project practitioners.

#### Case 2: Fabrication unit

The Fabrication Unit of NSG, located in two different industrial zones of Scotland, is involved in fabrication projects, including mostly manufacturing steel products for the oil and gas industry. This could be small pieces ranging from pipelines to underwater structures for the extraction of oil, or it could be the construction of process and/or compressor modules. The unit provides services mostly to offshore projects for new or, in technical jargon, “green fields.” These projects normally last from 1 to 7 months, with an average duration of 3 to 4 months.

The unit sometimes provides services to the OMI and Marine Service units in the case of a need to fabricate a special structure or equipment. Almost all of the projects are executed in the NSG’s facilities *onshore* and normally there is no need to send people offshore. Around 350 employees work in the Fabrication Unit and 60% of them are permanent staff. These are again mostly project managers, engineers and technical experts who work in the fabrication workshops. The remaining group of transient workers again comprises limited company contractors as the majority, and some agency workers. The unit receives HRM services from the group HR department mostly *via* online tools. However, there is an HR practitioner located in the unit, who is responsible mostly for the recruitment and coordination of HR practices.

#### Case 3: Marine services unit

The Marine Services Unit provides repair and maintenance services to drilling rigs, oil and gas ships, and vessels. These are mobile units involved in offshore drilling or field development projects. The ships normally carry the drilling rigs, and there are also various special ships for placing undersea vessels. While the majority of projects are implemented *offshore*, there are also projects executed completely at the shipyard. This depends on the scope of the work and the location of the ship or drilling facilities, the so-called “boat work.” Similar to the OMI Unit for marine projects, there is often a need to send people offshore for boat work. The duration of these projects varies from several days to 8 to 12 months, depending very much on the scope of the work. In a similar vein to the OMI Unit, the Marine Services Unit also makes use of many transient workers. While around 450 employees work in the unit, this can be increased for bigger projects. The majority of transient workers are limited company contractors; however, there are a few agency workers as well.

#### The north welding services (case 4)

The North Welding Services (hereafter NWS, also a pseudonym) is a specialist welding services contractor company that works within the oil and gas and energy industries in the North Sea region as well as overseas. The company is also based in Scotland but works not only in the United Kingdom but also in different European countries, West Africa and the Middle East. the NWS is involved in a range of different projects from *offshore* upstream to *onshore* downstream, i.e., refineries and petrochemical plants. This embraces working within *hook-up* (integration and installation of steel structures for offshore platforms) projects, piping projects, decommissioning of existing platforms and production facilities projects, and construction or expansion of refinery plants. Considering the diversity of its projects, the NWS is involved in projects of different lengths; this can be from 3 months, such as simple welding offshore, or onshore work up to 2 years in the full construction of a refinery or a hook-up project as well as the decommissioning of old assets offshore.

Despite the variety of NWS services, it provides these services through a small number of people. The company has 200 employees in total, which increases to as many as 500 during the peak time of projects. It also utilizes a mixture of permanent and temporary employment contracts. Only 30% of its employees work as permanent staff and 70% are employees who work temporarily. This includes Pay-As-You-Earn (PAYE) workers as the majority group of temporary employees, and limited company contractors (freelancers). The PAYE refers to employees who receive their wages weekly and their contract length is only 14 days; however, there is a possibility for renewal. Both groups have a direct contract with the company. The NWS has a small “Resource Planning” department that comprises only 4 practitioners, who are responsible for HRM activities. The department mostly supports different projects by assigning people to the project and managing their work cycles. The Resource Planning department provides its services to the company’s dispersed projects through close coordination with project managers and coordinators.

## Findings: HRM practices and the threefold structure of time, space and clients

### HRM practices

Several HRM practices are implemented in the NSG’s business units and the NWS. They are identified as recurrent situated HRM activities executed by multiple actors in the organization. People reflect their shared understanding of actions that need to be done and the steps that need to be followed for executing each practice and refer to policy and procedure documents that record and document these practices.

At NSG, the most important of these practices are recruitment, induction (socialization), training and competency management, and performance appraisal. However, other traditional administrative practices concerning the monitoring of time and attendance, leaves, dispute management and payroll, are also executed by the HR department. Although such practices are executed across the Group, each business unit customizes some of the practices in relation to its specific context (NSG-Recruitment Procedure). In this sense, there are salient differences, not only in the idiosyncratic nature of practices but also between units in executing them. The written HR procedures, forms and checklists maintain HR practices and “delineate the steps that should be followed for each practice” (NSG Recruitment Procedure). This is because each business unit has its procedure or performs the same practice differently. The following quote by the Group HR Manager explicates the point:

*“…the business units are individual. They are totally individual. Although they all work under one umbrella company, it has to be suitable for their own business, [for instance] the PRF [Personnel Request Form] may not be appropriate for their type of business activity and this means there's flexibility. One size will not fit across all business units...”*

Among all HR practices, recruitment, induction (socialization) and training, and competency management are highlighted as the main HRM practices in different units of NSG. For instance, the NSG HR Manager emphasizes that “*recruitment is probably one of the main drivers of the OMI within the HR team*. Moreover, inducting and training new employees for specific work contexts, particularly for offshore work, are crucial for the units as well, to ensure that people are competent to do the work because of the strict technical and safety standards of the industry. In this sense, the induction practice comprises several “technical and non-technical mandatory training courses that are essential for every new employee who joins the company” (NSG Training Procedure). The competency management practice focuses on the development of skills and competencies of current employees to ensure they maintain the right skillset for doing their jobs. The Head of OMI projects refers to this as the importance of “*the right people at the right place at the right time.”* The HR Manager Fabrication also refers to the same point:

*“Mainly, I mean most of my time is taken for recruitment. So, it's ensuring, obviously, that we have the right candidate on-site with the right compliances, that is, the right training ....”*

These HRM practices in NSG have been adapted to the specific contexts of the unit’s projects. The OMI unit has a rather unique recruitment process, since the majority of its employees are temporary offshore workers. On this premise, the process is started by filling in a detailed recruitment request form that clarifies almost all the required technical skills, certificates, permissions, and training courses regarding the job. The form, which is known as the Personnel Request Form (PRF), is the sole reference for the recruiter to search on a database of potential candidates in order to find a suitable person with the required skills and technical certificates. These candidates are temporary workers who have previous experience with the OMI’s projects, even for a very short period. Because of that, they are usually familiar with the Group’s and its clients’ policies and procedures, both technically and non-technically. The Fabrication Unit, which utilizes mostly permanent employees working onshore, follows the traditional steps for recruitment, i.e., “advertising the vacancy both internally and externally, shortlisting the CVs, interviewing the candidates” (HR Manager Fabrication). Interestingly, the PRF form is not used for recruitment in the Fabrication Unit and, in turn, the majority of candidates in the OMI Unit are not interviewed. The Group HR Manager articulates these differences:

*“We have two different ways of recruitment: offshore mostly for OMI and Marine, and then onshore mostly for Fabrication. So, for offshore recruitment, PRF is the Personnel Request Form. When project managers or a construction coordinator require a person to go offshore on a platform to do the scope of work, they put in a PRF... our onshore recruitment is at a slightly slower pace, it is very job description based [and] it is decided upon the relevancy of the role, and then it can be advertised... “*

While the Marine Services Unit resembles the OMI Unit in terms of the offshore working and utilizing a high number of transient workers, actually the recruitment practice depends on what is called *boat work:*

*“Basically, wherever the boat is we would provide, say a welder or a pipefitter. So, it is not classified as classic offshore. It’s called Boat Work. So, the Boat might be in the harbour where they work. So, [the recruitment process is] something in between. Whereas Sam [the offshore recruiter], he actually sends people to the platform and offshore oil rigs”* (HR Operations Advisor-offshore).

On this premise, the Marine Services Unit mostly utilizes a rather mixed recruitment approach, depending on where the actual job is to be performed. In a similar vein, findings reveal that induction and training practices are implemented slightly differently among the NSG’s units, specifically at the OMI due to its special context. For instance, the sequence of actions for induction is different or, sometimes, there is a need for additional steps in different units. For example, clients add their mandatory courses into the OMI’s induction practice (field notes – OMI). There is the same situation for the NWS as well.

At NWS, *resourcing* (recruitment) and what is called *rotation management* are the salient HRM practices. The Manager of the Resource Planning department explains the reasons for this as follows:

“…*the recruitment is always busy, particularly as we're running in quite remote parts of the world, so the process of getting somebody up here from you know Glasgow, Edinburgh, Spain to here is quite difficult, so it takes lots of time. But rotation management [is also important] because our clients enable their work on maybe a 4-week outlook, and I need to be constantly in contact with them to make sure that we have got more work that will come in from them and we have sufficient resources at work for that…”*

While the majority of NWS employees are PAYE employees and limited companies’ contractors as temporary workers, similar to the OMI and Marine Services units of NSG, the recruitment practice mostly focuses on selecting potential candidates from a database as a pool of candidates. The practice begins when the project manager sends the request with technical details and requirements to the resourcing team, then the resource coordinator starts searching for candidates on the database, specifically based on technical and professional qualifications. Surprisingly, here there is no typical job interview with the candidate, either, and “*the candidate needs only to pass a practical test on the NWS’s workshop*” (Resource Planning Manager). The findings reveal that similar to NSG, while the practice for recruiting transient workers is mostly the same among different projects, it differs between offshore and onshore projects. In this sense, there are additional steps for offshore recruitment:

“…*obviously for offshore you need to have your offshore survival, you need to have your offshore medical, there are various specific courses that you cannot go offshore without, and that’s the difference for us...”* (NWS-Resource planning coordinator)

Apart from this, the recruitment practices for non-project and office-based staff are totally different. For the office staff, the typical recruitment practice includes advertising the job, collecting and short-listing the CVs, conducting interviews and selecting the candidate. In a similar vein, the *rotation management* practice is different between onshore and offshore projects. The practice refers to activities associated with planning and managing the working schedules of employees who work on projects on a rotation basis, including logistic affairs, i.e., accommodation and transportation. These work cycles are different for offshore and onshore projects, e.g., 2 weeks on/2 weeks off for offshore vis-à-vis 3 weeks work/1 week rest for onshore and also depending a great deal on where the project is located.

“*Each project also has its rotation cycle that is agreed upon when awarding the contract, based on negotiation with labour unions and the legislation of the country of the project”* (Resource Planning Coordinator).

### The threefold structure: Temporality, space and clients

The findings reveal differences between the HR practices of NSG’s units in terms of the work location, relationship with clients, and, more importantly, temporal conceptions that are reflected in the pace of doing activities, which affect the performing of HRM practices. In NWS, these differences are generally identified between projects based on the location of projects.

For instance, in the OMI Unit, recruiting a permanent staff member follows different steps to the one-step recruitment of a transient worker; what is more salient, however, is the different temporalities among the actors of this unit, which is reflected in the fast pace and rhythm of actions for recruiting offshore workers. The OMI context is “*quite reactive, fast pace and ever-changing*” (Group HR Manager), requiring practices performed in a very short period. In this sense, actors’ temporal conceptions affect the timing and sequencing of actions in this unit. The following quote from the Group HR Manager vividly illustrates the fast pace of actions in the OMI context:

*“The recruitment part of the OMI business unit is quite diverse, very fast pace. You know, requests can come in at 9 o'clock in the morning for someone to get onto an offshore helicopter at 12:00. So, it is a very fast pace, then we have to get all the way through all of these elements before then, and there are various gates they have to pass through before they are eligible to go.”*

There is the same situation for induction and training practices in the OMI Unit as well. The way actors sense time and the temporal structure they shape, based on their temporality, reflect the pace of their actions. NSG’s HR operations advisor-offshore explicates a common example of the induction practice:

*“…if it’s a very quick turnaround, the person might have to do 13 courses plus the mandatory client induction, which means he cannot travel offshore. The person might come to us and say I do not have time, I have to travel up to Aberdeen to mobilize at 6:00 am...”*

In this respect, different units of NSG reflect the different rhythms of actions emanating from different temporalities of actors. The ways that actors conceive project deadlines, time horizons and time pressures in different units are not the same. For instance, while the pace of performing HRM practices at the Marine Services is relatively similar to the OMI Unit, it contrasts to the Fabrication Unit’s slower pace. The HR operations advisor-onshore illustrates this contrast regarding recruitment practice:

*“I would say [it takes] two to three weeks... you've got all your CVs and then you know you will find someone but it could take longer, if you just continue to interview and don't find the right person ...”*

In a similar vein, the rhythm of performing HRM practices for offshore projects in the NWS is faster than for onshore project show ever, as the Resource Planning manager indicates, this is very dependent on what clients want:

*“… for offshore we receive the [recruitment] request with short notice. It can be very, very, short notice ... all the projects can be different. Offshore does tend to be a bit more reactive....”*

Furthermore, the findings also show that the different temporal structures of actors, which are reflected in the fast pace of performing HRM practices, for instance, in the OMI and the Marine Services Unit, sometimes lead to deviating from defined steps and sequencing in procedures or, as the case informants say, “*changing the sequence of steps*” (NSG-Group HR Manager). For instance, it is common for inducting a new transient worker at NSG not to go through all the mandatory courses defined for the induction before going offshore because of a short time available. This is despite it being clearly underlined in the procedure (NSG-Training Procedure). The NSG HR operations advisor-offshore gives an example:

*“…if we say the man cannot go tomorrow, we won't let him go. [Then] the client might say we need him tomorrow. So, our project team would have to deviate and say, with the induction courses, okay do only this, this [and] this. Whereas if we can get them the next day, then we have more time, so we do not deviate from the procedure. So most of them occur due to time constraints. It is a very quick pace. [For instance] yesterday maybe 1:00 pm one of the clients said we need a welder today for 10:00 am...okay!!??…so it is very quick…”*

Moreover, the findings illustrate that the *physical location* where the work is carried out has an impact on HRM practices and how they are performed. Differences between NSG business units and projects in NWS show that the physical space is an underlying dimension that distinguishes HRM practices between them. For instance, at NSG, the HRM practices are reconciled with onshore and offshore working features, while each unit is dominated by one of these types as already explicated above. The HR roles and responsibilities are also organized around these categories.

*“… the onshore recruitment process is not the same as offshore recruitment. So, we have got two recruiters primarily concentrating on offshore recruitment, which is slightly different to when you do it for onshore recruitment because they are more permanent, there are fewer, far fewer transient [workers] in onshore …”* (Training and Competency Management Advisor)

While the OMI unit is a pure example of offshore working, as the main part of projects is done on platforms at *sea*, the work in the Fabrication Unit is done at the NSG facilities on *land*. The Marine Services unit, as has been indicated before, cannot easily be categorized by the *land-sea* dichotomy, and it is highly dependent on the location of the *boat*. The NSG HR Manager compares business units in this regard:

*“...Fabrication does not have an offshore contingent [workers] at all. They'll have sites [workers] but there are lots of facilities in different geographical locations… [the]Marine is probably the closest likeness; they do have people go offshore and people go to site ....”*

Notwithstanding the influence of the onshore-offshore dichotomy on the development and performance of HRM practices, findings illustrate how the geographical location of projects, particularly the host country of the project with its contextual properties also has a prominent role in shaping HRM practices. This is particularly salient in overseas projects of the NWS. The following quote from the Resource Planning coordinator presents an example:

*"… [For recruitment] in Norway, all of the clients in Norway have been sending inspectors to supervise the technical tests…so there is an additional step to get their approval.”*

The findings also reveal that different projects in all cases have different *relationships* with clients that affect HRM practices. The NSG Operation Manager compares business units in this regard:

*“...the OMI’s relationship [with clients] is a very close and very tight interface between our people and clients’ people because it's a wide installation…so often we're providing people to support the scope of work and they may be managed by the client overall, so because we're going on producing assets the level of collaboration is higher [therefore] the engagement of the client is higher... at the marine works you've taken the ship away from work into the harbor, into the shipyard. So usually, again the client will spend many months, maybe even a year sometimes planning the program of work that needs to be done…they’ll typically do that with some levels of input from us but it's not great. If you do the work on offshore platforms, perhaps in the OMI with their business, the interaction with clients is different also in the Fabrication business, that's more like you shopping for something, placing an order with the factory to make you something …clients have a small presence...”*

Following these inter-organizational relationships of different quality, findings illustrate how clients intervene in the development and implementation of HRM practices. However, the level of this intervention varies among business units and projects and depends strongly on project properties such as size and technical features. Based on this premise, clients from time to time impose their expectations and/or demands regarding HR practices. This generally occurs directly in daily interactions between the HR team and clients, for instance in the case of the offshore recruitment, and in some cases indirectly, *via* the project management team, for example in training and inducting transient workers at the NSG and / or during the practical skill test in the NWS. More importantly, clients constantly review HRM practices through their frequent audits, inspections and throughout the tender process. These interventions are more salient when there is a close relationship with clients due to the type of project. The following quote elucidates the role of clients in developing and executing HRM practices:

*“… the course [for the induction] is specific for clients. We have our induction course on the e-learning system, which is a common induction. It introduces NSG so that’s our induction, There is a client-specific induction as well. So, they provide us with their materials and what they want to see …”* (NSG-HR operations advisor offshore)

*“In the first step we adhere to the robust procedures that [the NSG HR Manager] introduced to the business and myself. We made robust procedures…so that when we were audited we could prove to the clients ... we could prove to the clients that the people we provide them with are competent* …” (NSG-offshore recruiter).

**Figure 2:**
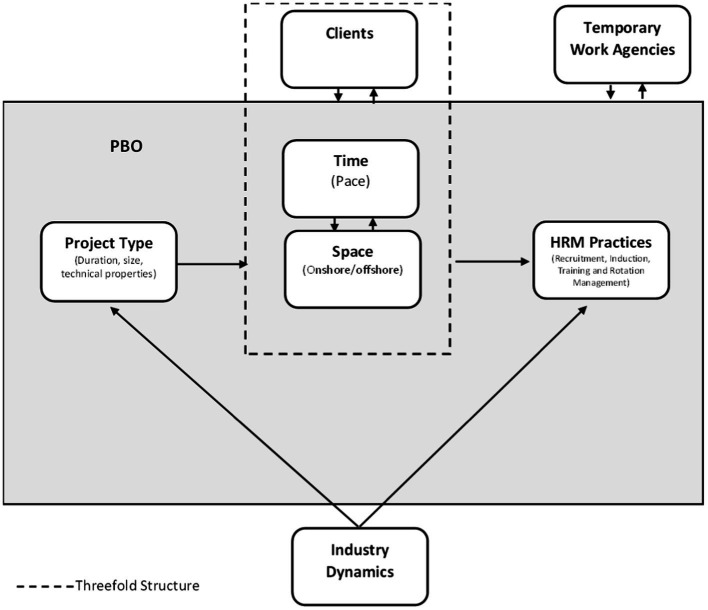
HRM practices in PBOs.

Moreover, findings reveal that these underlying dimensions are interrelated and highly intertwined as a threefold structure (see Figure 2). For instance, at the OMI Unit, where offshore working and short and small projects are salient, projects induce specific temporalities and close relationships with clients emanate from the specific physical space, the *sea*. By the same token, working on *land* with normally bigger and longer projects invokes specific temporalities and relationships with clients in the Fabrication Unit. Interestingly, the Marine Services Unit illustrates a highly dynamic relationship among these dimensions due to the importance of the *boat work.* The interaction of these dimensions of the tempo-spatial nexus is prominent in the following quote from the Head of OMI projects:

“*The difficulties that we have are due to the operational environment in which we work, the clients may demand people at very short notice, so if a client offshore has the operational need to change they can change our work...so, if we have got recruitment to do with 5 people in the next weeks, we have got people ready offshore on the platform [but] the client may simply not authorize the work, as they might be in a different operational mode. HR then have to deal with those people and they have to come out with that. So, this is because we are a very reactive organization. We sometimes ask people to work in a difficult situation, that means quite novel practices…"*

### Project types

Both NSG’s business units, as well as NWS, have been organized around different types of projects. Each type has different technical properties, duration and size that, as the findings reveal, have a pivotal role in the formation and shaping of HRM practices. For instance, while at NSG, the OMI Unit is mostly involved in *“small short-term repair projects on brownfields while most of the time the operation is ongoing during the execution of projects”* (the Head OMI Projects) “*the reactive nature of projects*, *their short durations and intensive time limitations*” (OMI construction manager) invoke particular temporalities and shape actors’ temporal structures as well as close relationships with clients. These project features determine the main space for executing the actual work, which, in this case, is on the platform at *sea*. By contrast, the fabrication projects, which are like “*placing an order with the factory”* (NSG- Operation Manager), require working on the facility on *land* with a particular temporality and relationship with clients. In a similar vein, the dynamic nature of projects in the Marine Services Unit induces different temporal conceptions, space and clients’ relationships that vary for each project. The NSG Operations Manager refers to the influence of characteristics of projects on HRM practices in different NSG units:

*“…The Fabrication [unit] is mainly focused on people working in factories on land, whereas the actual delivery of the OMI project work is technically done offshore. So, the qualifications and experience you need are much greater. You need various passes, specific clients may require specific training. So, the placement of people to work in the marine business in the shipyard and work in the fabrication business is simple and more straightforward because there are fewer qualifications needed and fewer client-specific requirements for a candidate to meet. So, the amount of work that they spend on recruiting and positioning people to work [offshore] is greater than that involved in just getting somebody to come and work in the factory...you need a survival course, you need specific client inductions...you need to go offshore, you need helicopter training, all these kinds of thing...”*

Likewise, hook-up and decommissioning offshore projects at the NWS, which are bigger and more durable than other types, invoke specific temporalities and relationships with clients that distinguish them from short-term onshore projects. The following quote about the relationship with clients from the NSW’s project manager highlights this point:

“*I think we need to have a pretty good relationship with clients while the job is running. Obviously, if you are on a longer scope of work you really need to have a shorter and closer relationship [with clients] you need to liaise with the client, stuff like that… For instance, in the Mariner [a hook-up project] we have a great relationship with the client, our scope in the Mariner grew, and it grew and we end up with more and more people on it…”*

### Industry dynamics

As stated before, the industry faces many swings, mainly due to fluctuations in the oil price (see Figure 1). The study reveals that these swings impact both the projects, in terms of duration, size and technical properties, and the HRM practices in all cases. With the high oil price, clients are more motivated to invest in new projects, whereas downturns lead to a reduction in projects and more focus on existing production plants. Apart from that, fluctuations in the oil price directly affect the volume of production (see again Figure 1) that technically impacts the operation of production units. On this basis, NSG business units, for instance, are directly affected by these dynamics. The Head of OMI projects explains this effect:

*“…It is just about cycles, cycles of the oil price. As soon as the oil price booms, the clients will want more work, there will be more people [and] we can afford to do more things... when it goes over into recession, things tend to go like this: The clients will not pay [and] cut the machinery savings…, as soon as the price goes up, it is like, how can I increase the value? I want bigger projects right now...the industry’s been in a flux situation, they have been in a big downturn in the last 10 years, so there has not been a lot of development. So, we are never quite sure if we can grow the business at a different level.*”

Besides, findings reveal that a recession, unsurprisingly, leads to reducing the number of employees and / or in some cases to delimiting the recruitment of particular types of employees in different business units. For instance, due to the recession in past years, both the Fabrication and OMI units “*had to go through a massive redundancy wave”* (HR Manager Fabrication) and the Fabrication Unit could not recruit any permanent staff for a long time (NSG-HR operations advisor-onshore). On this basis, due to the recession, the recruiting of temporary workers has been promoted by the firm. The NSW’s CEO also refers to the effect of industry dynamics on the employment modes:

*“…in the old days, you may have had a lot of people work for companies. So, companies would have a lot of employees, what we call staff employees, but through the change to the downturn, through economic recession [and] through phenomenal growth times within the industry, a lot of companies actually moved away from that, so they wanted to have the flexibility to be able to take only the key, core overhead people.”*

Moreover, the findings indicate, even more importantly, that the cyclical nature of the industry might lead to the suspension or elimination of particular HRM practices, such as the recruitment of apprentices. The following quote explicates these examples.

*“We have not taken apprentices since 2014 because of the downturn... So, this year, for the first time, we take apprentices...just, you know, a lot of changes due to the downturn. We took on 20 apprentices in 2014 and we have only taken 4 this time. One time, you know, everything in the oil industry was great...so it is related to the oil price. When the oil price is high we can recruit ...When it was not high, we were trying to keep our men and our jobs...”* (NSG-HR Operations Advisor-onshore).

## Discussion and conclusion

This research answers two questions: (1) *How are HRM practices formed in PBOs? (2) How does the tempo-spatial affect the formation of HRM practices in PBOs?* Concerning the first question, the results show vividly how project type and context drive HRM practices in PBOs. As the cases demonstrate, project characteristics, specifically their duration, size and technical properties, induce different temporalities as well as different work locations and inter-organizational relationships that impact the formation and adoption of HRM practices. As far as project context is concerned, industry dynamics also affect project properties, specifically the duration and size of projects, and have a direct impact on HRM practices by promoting and / or delimiting particular practices.

In response to the second research question, while organizational actors constantly form and re-form different temporal structures and temporalities that shape the temporal pace of their ongoing actions (Dahlgren and Söderlund, 2001; Orlikowski and Yates, 2002; Braun and Lampel, 2020), the findings illustrate how different temporalities in different projects or parts of PBOs are an integral part of the recurrent situated actions of PBOs’ actors and are reflected in the “duration, sequence, timing, frequency” (Zerubavel, 1981) and particularly the pace of HRM practices. For instance, as the offshore recruitment in the OMI clearly shows, the ways that PBOs’ actors conceive time and their own temporalities play a salient role, not only in developing and adapting HRM practices in terms of the sequence and type of actions, but also in adjusting the internal rhythm of actions. In addition, the findings not only reveal the salient role played by the physical space of projects (van Marrewijk and Smits, 2014) in forming HRM practices, but also shed light on how the interaction between time and space affects HRM practices in the project context (Maaninen-Olsson and Müllern, 2009).

Against these two backgrounds, HRM practices unfold and take shape at the intersection or the *edge* of time, space and relationships with clients, and are constantly adapted to a threefold structure. Figure 2 depicts the model that has emerged from the findings.

This research thus contributes to HRM and to project management research as follows. First, this research, adopting a practice-based perspective to study HRM (Watson, 2004; Vickers and Fox, 2010; Ripamonti and Scaratti, 2011) and projects (Manning, 2008; Blomquist et al., 2010; Clegg et al., 2018), unveils the role of project and contextual properties in shaping HRM practices in PBOs, a so-far under-researched topic. This corroborates the finding that context matters a lot when organizing projects in general and PBOs in particular (Grabher, 2002; Sydow and Staber, 2002; Engwall, 2003; Sydow et al., 2004; Manning, 2008). While at the project level, project-specific features like project length, size and technical properties affect HRM practices through the indicated tripartite structure, at the field level, industry dynamics have an important influence on projects and organizational actions (see Figure 2).

Furthermore, in contrast to previous research on HRM in PBOs, which mostly ignores the effect of project-specific properties on HRM practices (Turner et al., 2008; Huemann, 2010, 2015; Keegan et al., 2018; Samimi and Sydow, 2021), this research illuminates how particular project features, together with industry dynamics, contribute to forming and shaping HRM practices. Second, more importantly, the research illuminates the salient role of *temporality* in regard to temporal conceptions (Zerubavel, 1981; Emirbayer and Mische, 1998; Ancona et al., 2001b; Orlikowski and Yates, 2002; Braun and Lampel, 2020) and “temporal tensions” (Dahlgren and Söderlund, 2001; Stjerne et al., 2019) in shaping a PBO’s practices. In this sense, while traditionally PBOs have been analyzed through the “temporary-permanent dichotomy” (Sahlin-Andersson and Söderholm, 2002), surprisingly, the cases indicate that PBOs’ actors rarely refer to this dichotomy and the related tension to explicate the organizations’ activities. Instead, what is foregrounded and more prominent is the different temporalities of actors, which are reflected in the pace of actions in different parts of PBOs. While the temporal tensions have been studied at different analytical levels such as “at the intersection of markets and human development programs” (Reinecke and Ansari, 2015) or inter-organizational projects (Dahlgren and Söderlund, 2001; Stjerne et al., 2019), this research elucidates their pivotal role at the firm level, in shaping organizational practices in PBOs.

Third, while all human actions are bounded in time and space (Giddens, 1984) the research sheds light on how spaces matter in organizational actions in general and HRM practices in PBOs in particular. This is important because traditionally, time has received much more appreciation in temporary organization studies (Bakker, 2010; Bakker et al., 2016; Burke and Morley, 2016; Braun and Lampel, 2020) than space (Maaninen-Olsson and Müllern, 2009; van Marrewijk and Smits, 2014). Considering space, however, offers fertile ground to scrutinize the spatial aspects of the context in which temporary organizations are embedded and their impact on organizational actions such as HRM practices. Additionally, while the role of interaction between time and space and the tempo-spatial nexus in shaping practices in the project context is under-researched, this research sheds light on how the tempo-spatial nexus affects HRM practices in conjunction with the inter-organizational relationship as a dynamic threefold structure (Maaninen-Olsson and Müllern, 2009; Stephenson et al., 2020).

Finally, the study shows how inter-organizational relationships, in this case relationships with clients, are important to HRM practices in PBOs. Although these relationships have been studied mostly at the project network level (Windeler and Sydow, 2001; Sydow and Staber, 2002) this research vividly illustrates their direct effects at the organizational level.

In line with these contributions, this study has implications for future research. While the research elucidates the effects of a threefold structure of temporality, space and relationships with clients on shaping HRM practices in general, in the project context future research can scrutinize the effects of this structure on particular HRM practices, for instance on employment or competency development practices and can investigate their peculiarities in the project context. In a similar vein, to elucidate further how the presented tripartite structure affects not only HRM practices but also HRM configurations, future studies can address how it impacts shaping and implementing HRM strategies in the project context. Moreover, while this research shows the role of project properties, specifically, size, duration, and technical properties of projects in shaping HRM practices future research can illuminate how each of these properties impacts HRM in the project context.

Apart from these implications, the research also has limitations. Lack of similar studies of HRM practices and/or practices in the project management and HRM scholarly fields, the specific geographical conditions of the cases and qualitative approach of the research, affect the generalizability of the result. Furthermore, while the research focuses on the impact of structural properties of projects on shaping HRM practices, it does not explore the role of other relevant contextual elements, e.g., institutional elements in the formation of HRM practices in the context of project.

## Data availability statement

The raw data supporting the conclusions of this article will be made available by the authors, without undue reservation.

## Author contributions

The author confirms being the sole contributor of this work and has approved it for publication.

## Conflict of interest

The author declares that the research was conducted in the absence of any commercial or financial relationships that could be construed as a potential conflict of interest.

## Publisher’s note

All claims expressed in this article are solely those of the authors and do not necessarily represent those of their affiliated organizations, or those of the publisher, the editors and the reviewers. Any product that may be evaluated in this article, or claim that may be made by its manufacturer, is not guaranteed or endorsed by the publisher.
